# ASO Author Reflections: Outcomes of Hepatic Artery-Based Therapies and Systemic Multiagent Chemotherapy in Unresectable Colorectal Liver Metastases: A Systematic Review and Meta-analysis

**DOI:** 10.1245/s10434-024-15309-6

**Published:** 2024-04-28

**Authors:** Kavin Sugumar, Lee M. Ocuin

**Affiliations:** 1 Department of Surgery, Tulane University School of Medicine, New Orleans, LA USA; 2 Division of Surgical Oncology, Department of Surgery, University Hospitals Cleveland Medical Center, 11100 Euclid Avenue, Cleveland, OH 44106 USA

## Past

Colorectal cancer (CRC) most commonly spreads to the liver, and approximately 80% of patients have technically unresectable disease burden at diagnosis.^1^ At present, these patients receive systemic multiagent chemotherapy, sometimes in combination with liver-directed therapies.^2^ Despite advances in systemic chemotherapy and molecular targeted therapy, modern first-line standard-of-care chemotherapy regimens very rarely facilitate conversion to resection, and patients who proceed to receive liver-directed therapy do so in second- or third-line therapy. Numerous locoregional therapies have been investigated over the last two decades including hepatic artery infusion therapy (HAI), transarterial chemoembolization (TACE), and transarterial radioembolization (TARE). Currently, there exists no consensus on the long-term effectiveness of various liver-directed therapies compared with modern multiagent chemotherapy in patients with unresectable colorectal liver metastasis (UCRLM).

## Present

Historically, studies have shown that HAI alone is better than single-agent systemic chemotherapy.^3^ However, modern HAI is usually given concomitantly with systemic chemotherapy (HAI-S) in most cancer centers. No prospective studies to date have compared HAI-S with modern multiagent chemotherapy. Similarly, a meta-analysis from 2015 comparing TACE, TARE, and HAI used historical cohorts from early experience of liver-directed therapies and failed to show a difference in long-term outcomes.^4^

To overcome the previous limitations and to provide a comprehensive update of existing evidence, a systematic review and meta-analysis was performed of studies including patients with UCRLM treated with one of the five treatment arms: HAI-S (HAI with systemic therapy), TACE with systemic chemotherapy (TACE-S), TARE with systemic chemotherapy (TARE-S), doublet chemotherapy (FOLFOX, FOLFIRI), and triplet chemotherapy (FOLFOXIRI). Outcomes included overall survival (OS), progression-free survival (PFS), conversion-to-resection rate (CTR), and response rate (RR).^5^ On pooled analysis, the triplet chemotherapy regimen FOLFOXIRI seemed to outperform other arms; however, on subgroup analysis for patients receiving first-line therapy and without extrahepatic disease, the HAI-S arm had comparable outcomes to FOLFOXIRI and appeared to outperform the doublet regimens. Excluding studies with pretreated and extrahepatic disease, in the HAI-S arm more than 50% of patients survived 3 years and up to 50% did not show disease progression until 1–2 years after initiating therapy. TACE-S and TARE-S were associated with poor survival, with more than two-thirds of patients dying at the end of 2 years. Despite the promising outcomes of FOLFOXIRI, given the associated higher toxicity profile, inclusion of patients with resectable CRLM in several studies, and concurrent use of biological agents, broader utilization in patients with UCRLM needs to be further evaluated.

## Future

The treatment paradigm for UCRLM continues to evolve rapidly. Most of the current evidence for liver-directed therapies comes from single-institution retrospective studies. Our meta-analysis shows that HAI-S and multiagent chemotherapy are effective in treating UCRLM. Randomized clinical trials in patients with comparable disease burden and lines of therapy are required to compare the long-term outcomes of HAI-S to systemic multiagent chemotherapy. The ECOG-ACRIN trial (EA2222/The PUMP Trial; NCT05863195), which is currently enrolling patients, will help address this question.
